# Risk prediction model for cognitive frailty in older adults with diabetes: a systematic review and meta-analysis

**DOI:** 10.3389/fendo.2026.1794278

**Published:** 2026-04-10

**Authors:** Yongyuan Luo, Heng Duan, Tingyu Yang, Zhongxi Hong, Chen Huang, Xiya Xiong, Xuemei An

**Affiliations:** 1 College of Nursing, Chengdu University of Traditional Chinese Medicine, Chengdu, China; 2 Affiliated Hospital of Chengdu University of Traditional Chinese Medicine, Chengdu, China; 3 Chengdu University of Traditional Chinese Medicine Affiliated Hospital Deyang Hospital, Deyang, China

**Keywords:** cognitive frailty, diabetes mellitus, meta-analysis, risk prediction model, systematic review

## Abstract

**Objective:**

Cognitive frailty (CF) represents a significant geriatric issue closely linked to diabetes. Although multiple CF risk prediction models exist for older adults with diabetes, their methodological quality and clinical utility remain unclear. This systematic review evaluates the predictive performance and risk of bias of existing models to provide inform clinical practice.

**Methods:**

A systematic search was conducted in PubMed, Embase, Web of Science, Cochrane Library, CINAHL, Sinomed, CNKI, and Wanfang from inception to September 2025. Two researchers independently performed literature screening, data extraction, and quality assessment. Study and model characteristics were summarized descriptively; pooled AUC values were analyzed using Stata 17.0. PROBAST was used to evaluate risk of bias and applicability.

**Results:**

Eight studies involving 2,947 diabetic patients were included. CF prevalence ranged from 12.1% to 40.0%. Predictors encompassed sociodemographic, disease-related, psychological, and lifestyle factors, with age, depression, diabetes duration, nutritional status, and regular exercise being most frequently reported. The models showed good discrimination (AUC: 0.790-0.975) but exhibited high overall bias risk.

**Conclusion:**

Existing CF prediction models demonstrate acceptable discrimination but are limited by high bias risk and poor applicability. Future research should prioritize developing rigorously designed models with multicenter external validation to enhance prediction accuracy.

The study was reported in accordance with the Preferred Reporting Items for Systematic Reviews and Meta-analyses (PRISMA) guidelines (17). The study protocol was registered on PROSPERO (CRD420251054250).

**Systematic Review Registration:**

https://www.crd.york.ac.uk/PROSPERO/view/CRD420251054250, identifier CRD420251054250.

## 1 Introduction

The accelerating global population aging has rendered diabetes in the elderly a significant public health challenge worldwide (1). As reported in the International Diabetes Federation (IDF)’s *Diabetes Atlas 2021 (10th edition)*, approximately 537 million adults aged 20–79 years had diabetes globally, corresponding to a prevalence of 10.5%, with this figure rising markedly to 24.0% among those aged 75–79 years (2). In this context, research targeting this population should not only focus on disease control and delaying disability but also embrace the core concept of “healthy aging,” aiming to maintain and enhance positive outcomes such as quality of life, functional independence, and social participation (3, 4).

Frailty and cognitive impairment represent major research priorities in geriatrics (5). Frailty is a geriatric syndrome characterized by reduced physiological reserve across multiple systems, which manifests as increased vulnerability to stressors and elevated risk of adverse health outcomes (6). Cognitive impairment denotes objectively measured deficits in at least one cognitive domain (e.g., memory, attention, executive function), often considered a prodromal stage of dementia (7). Given their close interrelationship and frequent co-occurrence, the International Consensus Group proposed the concept of cognitive frailty (CF) in 2013, defined as the co-occurrence of physical frailty and cognitive impairment (with a clinical dementia rating [CDR] score of 0.5), excluding Alzheimer’s disease (AD) or other dementias (5). CF constitutes an intermediate transitional state between normal aging and severe disability (e.g., physical disability or dementia) (5) and serves as a critical predictor of adverse outcomes in older adults (8).

Accumulating evidence demonstrates significant associations between diabetes and both frailty and cognitive impairment. Older adults with diabetes exhibit a 2.18-fold higher prevalence of frailty and a 1.5-fold increased risk of cognitive impairment relative to non-diabetic individuals (9, 10). Notably, CF exerts additive effects on adverse outcomes (11): diabetic patients with concomitant CF face substantially elevated risks of dementia, falls, injuries, disability, and mortality compared to those with isolated frailty or cognitive deficits (12–14). Given these compounded risks, early identification of at-risk individuals is critical to prevent functional decline and associated adverse health outcomes.

Ruan et al. (15) proposed the concepts of reversible cognitive frailty (RCF) and potentially reversible cognitive frailty (PRCF) in 2015, suggesting that timely intervention on modifiable risk factors may reverse certain CF cases. Considering this potential reversibility, the development and validation of scientifically robust risk prediction models are urgently needed to enable proactive identification of high-risk patients.

Despite increasing efforts to develop risk prediction models for CF in older adults with diabetes, substantial methodological heterogeneity persists across studies, particularly in predictor selection, statistical modeling approaches, and validation procedures. Moreover, most existing models lack external validation and adequate assessment of clinical applicability, limiting their translation into routine geriatric care. Consequently, the methodological quality and predictive reliability of currently available models remain uncertain (16).

This study consolidates evidence via systematic review and critical appraisal, thereby mitigating inherent biases in individual studies while comprehensively evaluating predictive performance. This approach provides evidence-based guidance for clinicians to select, optimize, or develop clinically applicable risk assessment tools and intervention strategies. Ultimately, this work contributes to the early identification and management of CF in this vulnerable population, supporting the broader goal of promoting healthy aging.

## 2 Methods

The study was reported in accordance with the Preferred Reporting Items for Systematic Reviews and Meta-analyses (PRISMA) guidelines (17). The study protocol was registered on PROSPERO (CRD420251054250).

### 2.1 Problem formulation

To enhance the clarity and transparency of the review question and eligibility criteria, the PICOTS framework was adopted (18). This framework facilitates a structured approach to defining the population, prediction models, outcomes, timing, and settings, enabling systematic consideration of these key elements during study selection and evidence synthesis. Moreover, by explicitly aligning each study’s design characteristics, the use of PICOTS helped clarify sources of heterogeneity across studies and supported a more rigorous comparison and synthesis.

P (Population): Older adults with diabetes;

I (Index prediction model): Risk prediction model for cognitive frailty;

C (Comparator): Not applicable;

O (Outcome): Presence of cognitive frailty;

T (Timing): No restrictions were applied regarding timing;

S (Setting): In a hospital, community or nursing home.

### 2.2 Literature inclusion and exclusion criteria

#### 2.2.1 Inclusion criteria

(1) Population: Older adults (≥ 60 years) with clinically confirmed type 1 or type 2 diabetes (2). Intervention of interest: Studies focused on the development, validation, or updating of risk prediction models for CF in older adults with diabetes (3). Study design: Cohort studies, case-control studies, or cross-sectional studies (4). Outcome: CF confirmed using validated assessment tools (5). Publication language: English or Chinese.

#### 2.2.2 Exclusion criteria

(1) Studies that solely analyzed risk factors without developing or validating prediction models (2). Models constructed with two or fewer predictors/variables (3). Conference abstracts, letters, reviews, meta-analyses, commentaries, or studies not presenting original research data (4). Duplicate publications (5). Studies where the full text was unavailable or contained insufficient data for extraction/analysis.

### 2.3 Search strategy

A systematic search was performed in databases including PubMed, Embase, Web of Science, the Cochrane Library, CINAHL, Sinomed, CNKI, and Wanfang, covering publications up to September 2025. The search strategy combined MeSH terms and free-text keywords, incorporating terms such as “Diabetes Mellitus,” “Diabetes Mellitus,” “Diabetic,” “Cognitive frailty,” “Predict*,” “Predict* model,” “Nomogram,” “Area under curve,” etc. The search strategy was adjusted according to the specific characteristics of each database. Additionally, the reference lists of relevant studies were manually screened (the search strategy for each database is described in **Supplementary Table 1**).

### 2.4 Literature screening and data extraction

Two researchers independently performed literature screening, data extraction, and cross-validation according to the inclusion and exclusion criteria. Inter-rater discrepancies were resolved through discussion, persistent disagreements required third-party arbitration by a senior researcher.

The process encompassed (1): Using NoteExpress software for reference management and duplicate removal (2). Excluding obviously irrelevant studies after reviewing titles and abstracts (3). Conducting a critical review of the full texts of eligible studies and assessing their methodological quality (4). Documenting specific reasons for exclusion (5). Extracting data via a predefined form, which included: (i) basic study information (first author, publication year, region, study design, population, assessment tool, sample size); (ii) prediction model details (variable selection, handling of missing data, modeling methods, model presentation format, final predictors, validation type, and performance metrics).

### 2.5 Methodological quality assessment

Two investigators independently assessed the risk of bias and applicability of the included studies using the Prediction model Risk Of Bias Assessment Tool (PROBAST) (19). Disagreements were resolved through discussion or consultation with a third researcher.

Bias risk was assessed across four domains (participants, predictors, outcomes, and analysis) using 20 specific signaling questions. Applicability was concurrently evaluated for participants, predictors, and outcomes. Each item was judged as “Yes,” “Probably Yes,” “Probably No,” “No,” or “No Information,” with domains categorized as “Low risk” if all items were rated “Yes” or “Probably Yes”; “High risk” if any item received “No” or “Probably No”; and “Unclear” when insufficient information led to “No Information” ratings. The overall risk of bias was subsequently determined by synthesizing domain-level assessments: “Low” when all domains achieved “Low risk”; “High” if any domain was rated “High risk”; and “Unclear” when unclear domains coexisted with otherwise low-risk components.

### 2.6 Statistical analysis

Descriptive analytical methods were used to summarize the fundamental characteristics of the included studies and prediction models. Quantitative synthesis of the area under the curve (AUC) was conducted using Stata 17.0. Heterogeneity across models was assessed using the Q-test and I² statistic. A fixed-effects model was applied when I² ≤ 50% and P > 0.1, suggesting low heterogeneity. In cases where I² > 50% and P ≤ 0.1, indicating substantial heterogeneity, a random-effects model was employed, and subgroup analyses were performed to explore potential sources of heterogeneity. Sensitivity analysis was conducted using a leave-one-out approach to evaluate the robustness of the pooled estimates. Publication bias was evaluated through funnel plots and Egger’s test, with P > 0.05 suggesting a low likelihood of publication bias.

## 3 Results

### 3.1 Literature screening process

**Figure 1:**
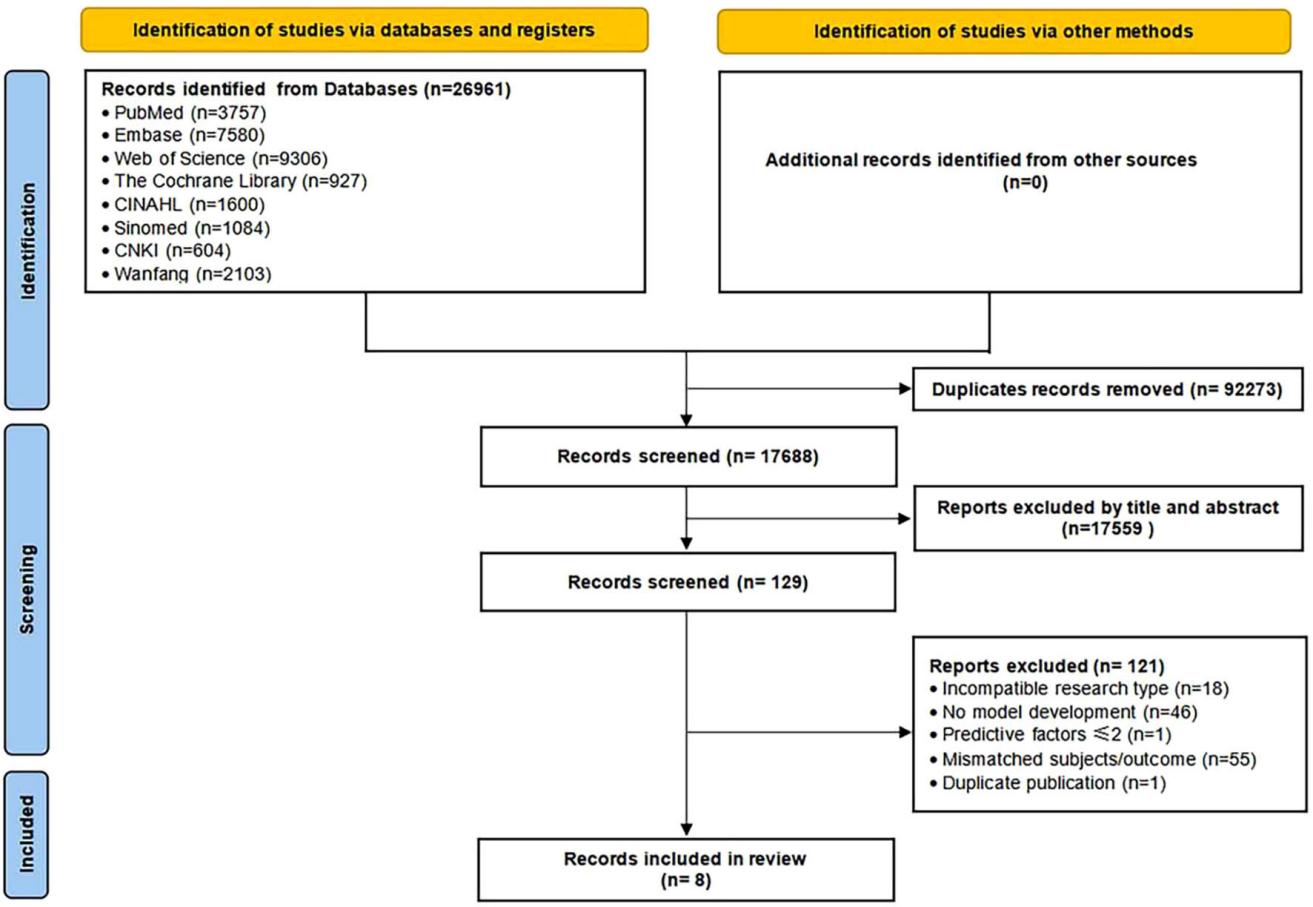
Literature screening process and results.

According to the inclusion and exclusion criteria, a comprehensive search was conducted across eight Chinese and English databases, yielding a total of 26,961 articles. After duplicate removal, initial screening, and re-screening, a final set of eight articles was included for analysis (20–27). The literature screening process and results are illustrated in **Figure 1**.

### 3.2 Characteristics of included studies

Eight observational studies published between 2023 and 2025, comprising seven cross-sectional (20–26) and one retrospective cohort (27), were conducted in China. The study population predominantly consisted of 2,947 older adults with type 2 diabetes recruited from community or hospital settings. CF was assessed using combinations of physical frailty scales (Fatigue, resistance, ambulation, illness, loss of weight scale [FRAIL]; Frailty Phenotype [FP]) and cognitive function assessments (Montreal Cognitive Assessment [MoCA]; CDR). The overall reported CF prevalence ranged from 12.1% to 40.0%. Detailed characteristics of the included studies are presented in **Table 1**.

**Table 1:** The characteristics of included studies (n = 8).

Included study	Region	Study design	Study population	CF assessment tool	Sample size		
					Modeling/validation group (n)	Events (n)	Incidence (%)
Deng, 2023 (20)	China, Guangzhou	Cross-sectional study	Patients with diabetes aged ≥60 years	①FP ②MoCA	221/94	61/221 26/94	27.6 27.7
Qian, 2025 (21)	China, Jinzhou	Cross-sectional study	Older adults with type 2 diabetes	①FRAIL ②MoCA	300/130	132	30.7
Du, 2024 (22)	China, Zhengzhou	Cross-sectional study	Community-dwelling older adults with type 2 diabetes	①FP ②MoCA+CDR	527	64	12.1
Meng, 2025 (23)	China, Shenzhen	Cross-sectional study	Older adults with diabetes	①FRAIL ②MoCA	370/138	88/370 29/138	23.8 21.0
Wang, 2023 (24)	China, Shanghai	Cross-sectional study	Patients with type 2 diabetes aged ≥60 years	①FP ②MoCA+CDR	262	85	32.4
Liu, 2024 (25)	China, Tianjin	Cross-sectional study	Older adults with type 2 diabetes	①FP ②MoCA+CDR	338/145	68/338 30/145	20.1 20.7
Wang, 2025 (26)	China, Sichuan	Cross-sectional study	Hospitalized older adults with type 2 diabetes	①FP ②MoCA+CDR	152/50	60/152 20/50	39.4 40.0
Liu, 2024 (27)	China, Anhui	Retrospective cohort study	Advanced-age patients with type 2 diabetes	①FRAIL ②MoCA+CDR	132/88	82	32.3

### 3.3 Characteristics of prediction model development

**Figure 2:**
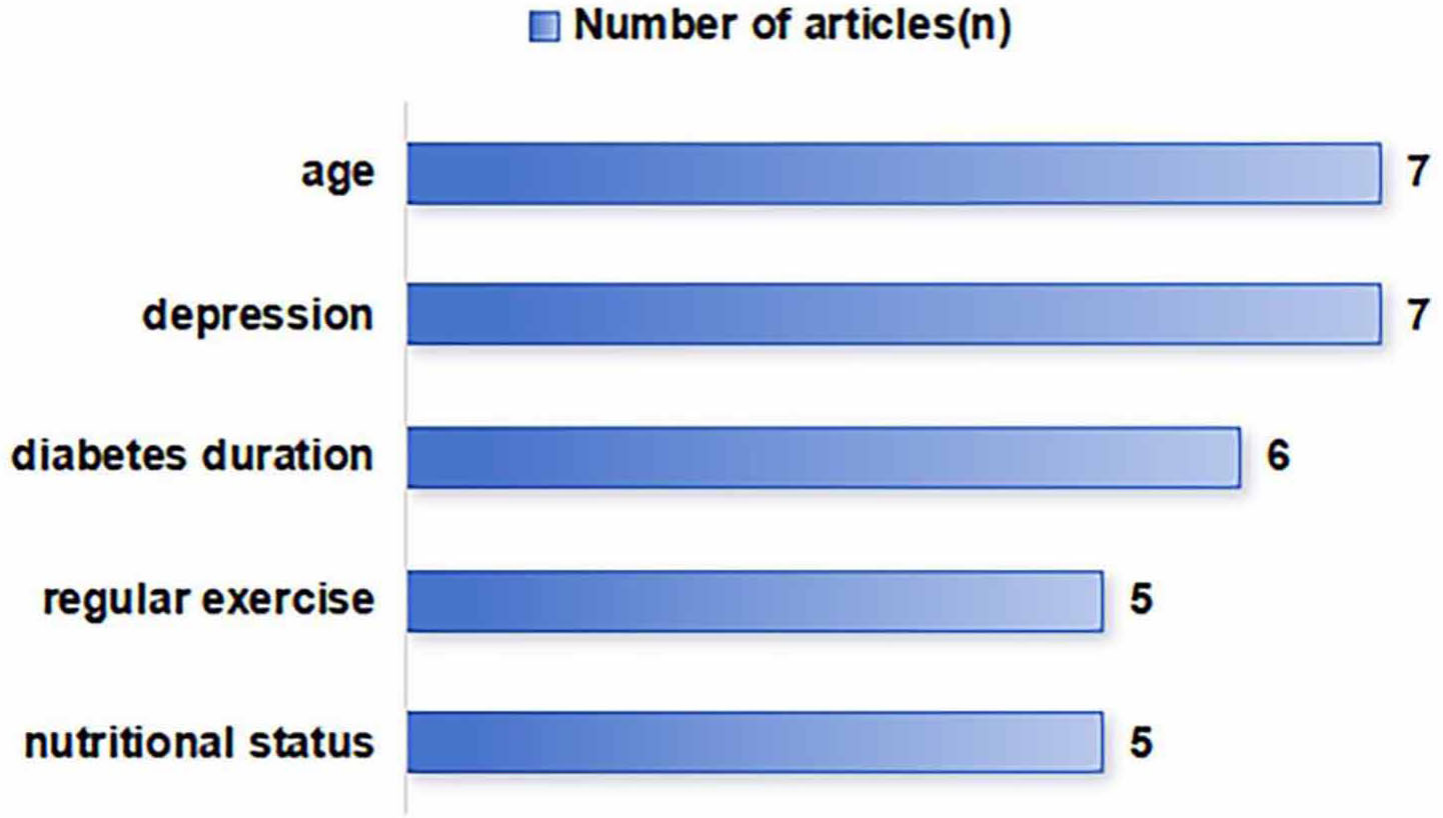
Predictor frequency distributions.

The number of candidate predictors in the models ranged from 11-26. Qian et al. (21) employed LASSO regression followed by multivariable regression; Wang et al. (26) used LASSO regression combined with univariable analysis; Deng et al. (20) utilized a sequential approach of univariable analysis, LASSO regression, and multivariable regression; and the remaining five studies (22–25, 27) adopted univariable analysis combined with multivariable regression for variable selection. For missing data handling, four studies (20, 21, 23, 24) excluded questionnaires with incomplete values during analysis; one study (27) restricted inclusion to patients with complete clinical records; one study (25) implemented on-site verification to minimize missing data; and two studies (22, 26) did not report missing data protocols. Regarding model development, one study (26) employed a decision tree model; and the remaining studies utilized logistic regression models predominantly visualized through nomograms (20–25), except for one formula-based model (27). Final models incorporated 4–8 independent predictors, with the five most frequent being: age, depression, diabetes duration, nutritional status, and regular exercise. Details of model development are summarized in **Table 2**, and the distribution frequency of the main predictors is illustrated in **Figure 2**.

**Table 2:** The characteristics of prediction model development (n = 8).

Included study	Variable selection		Missing data handling	Modeling method	Presentation format	Final predictor	
	Candidate variable (n)	Screening method				Predictor count (n)	Specific predictor
Deng, 2023 (20)	23	Univariate + LASSO + Multivariate regression	Direct removal	LR	Nomogram	6	Age, Intellectual Activity, Albumin, Calf Circumference, Depression, Diabetes Duration
Qian, 2025 (21)	22	LASSO + Multivariate regression	Direct removal	LR	Nomogram	7	Age, Physical Activity, HbA1c, Diabetes Duration, Nutritional Status, ADL, Depression
Du, 2024 (22)	19	Univariate + Multivariate regression	Not mentioned	LR	Nomogram	7	Age, Living Alone, Depression, Nutritional Status, Sleep Quality, HbA1c, Regular Exercise lesions
Meng, 2025 (23)	26	Univariate + Multivariate regression	Direct removal	LR	Nomogram	6	Regular Exercise, HbA1c, Diabetes Duration, Nutritional Status, Hypoglycemia History, CKD History
Wang, 2023 (24)	11	Univariate + Multivariate regression	Direct removal	LR	Nomogram	8	Gender, Age, Education Level, Regular Exercise, Memory, Nutritional Status, ADL, Depression
Liu, 2024 (25)	24	Univariate + Multivariate regression	On-site correction	LR	Nomogram	6	Age, Regular Exercise, Diabetes Duration, HbA1c, Depression, Nutritional Status
Wang, 2025 (26)	11	LASSO + Univariate regression	Not mentioned	Decision Tree	Decision Tree	5	Age, Living Arrangement, Depression, Diabetes Duration, Weekly Exercise Frequency
Liu, 2024 (27)	16	Univariate + Multivariate regression	Complete case analysis	LR	Formula	4	Age, Diabetes Duration, Sleep Duration, Depression

ADL, Activities of Daily Living; CKD, Chronic kidney disease; LR, Logistic regression; HbA1c, Glycated hemoglobin.

### 3.4 Model validation and performance evaluation

In terms of model validation, only two studies (21, 23) conducted both internal and external validation, while the remaining six (20, 22, 24–27) reported only internal validation methods. Regarding model performance, all models reported AUC values ranging from 0.790 to 0.975. Calibration was assessed by five studies (20, 23–25, 27) using a combination of calibration curves and Hosmer-Lemeshow (H-L) tests; two studies evaluated calibration solely through the H-L test (22) or calibration curves (21), respectively, while one study (26) did not report calibration methodology. Additionally, clinical utility was evaluated using decision curve analysis (DCA) in four studies (20, 21, 25, 27). Comprehensive validation and performance metrics are detailed in **Table 3**.

**Table 3:** The validation and performance metrics of prediction model (n = 8).

Included study	Validation method		Model performance				Evaluation of clinical effectiveness
	Internal validation	External validation	AUC(95%CI)	Sensitivity	Specificity	Calibration method	
Deng, 2023 (20)	Random split (7:3) + Bootstrap sampling	–	Modeling: 0.866 (0.809-0.922) Validation: 0.821 (0.716-0.927)	–	–	H-L test + Calibration curve	DCA
Qian, 2025 (21)	Bootstrap sampling	Time split	Modeling: 0.846 (0.799-0.893) Validation: 0.824 (0.747-0.902)	–	–	Calibration curve	DCA
Du, 2024 (22)	Bootstrap sampling	–	0.790(0.728-0.852)	–	–	H-L test	–
Meng, 2025 (23)	Bootstrap sampling	Time split	Modeling: 0.838 (0.789-0.887) Validation: 0.895 (0.827-0.964)	Modeling: 79.8% Validation: 80.7%	Modeling: 76.1% Validation: 86.2%	H-L test + Calibration curve	–
Wang, 2023 (24)	Bootstrap sampling	–	0.897(0.844-0.949)	–	–	H-L test + Calibration curve	–
Liu, 2024 (25)	Random split (7:3) + Bootstrap sampling	–	Modeling: 0.866 (0.839-0.932) Validation: 0.875 (0.809-0.941)	Modeling: 72.1% Validation: 70.0%	Modeling: 93.3% Validation: 86.1%	H-L test + Calibration curve	DCA
Wang, 2025 (26)	Random split (7:3)	–	Modeling: 0.908 (0.857-0.957) Validation: 0.868 (0.764-0.972)	Modeling: 76.7% Validation: 70.0%	Modeling: 92.4% Validation: 90.0%	–	–
Liu, 2024 (27)	Random split (3:2)	–	Modeling: 0.975 (0.955-0.985) Validation: 0.939 (0.943-0.972)	Modeling: 95.6% Validation: 92.9%	Modeling: 94.8% Validation: 81.1%	H-L test + Calibration curve	DCA

AUC, Area under the curve; DCA, Decision curve analysis; H-L test, Hosmer-Lemeshow test.

### 3.5 Risk of bias and applicability assessment of included studies

Eight studies were rated at high overall risk of bias. Seven demonstrated good overall applicability, while one study showed poor applicability. Detailed information is presented in **Table 4**.

**Table 4:** The risk of bias and applicability assessment of included studies (n = 8).

Included study	Risk of bias				Applicability			Overall	
	Participants	Predictors	Outcome	Analysis	Participants	Predictors	Outcome	Risk of bias	Applicability
Deng, 2023 (20)	+	+	+	–	+	+	+	–	+
Qian, 2025 (21)	+	+	–	–	+	+	+	–	+
Du, 2024 (22)	+	+	–	–	+	+	+	–	+
Meng, 2025 (23)	+	+	–	–	+	+	+	–	+
Wang, 2023 (24)	+	+	–	–	+	+	+	–	+
Liu, 2024 (25)	+	+	–	–	+	+	+	–	+
Wang, 2025 (26)	+	+	+	–	+	+	+	–	+
Liu, 2024 (27)	–	+	–	–	–	+	+	–	–

+, Low risk; -, High risk; ?, Unclear.

#### 3.5.1 Risk of bias

(1) Participants domain: one retrospective study (27) was rated high-risk primarily due to potential selection bias from data integrity and accuracy concerns inherent to retrospective designs. The remaining studies (20–26) utilized appropriate data sources with clearly defined inclusion criteria and were rated low-risk. (2) Predictors domain: one study (27) was rated high-risk because it: (i) lacked standardized measurement methods for key predictors (e.g., dichotomized sleep duration at eight hours; used unvalidated clinical assessments for hearing impairment, risking subjective bias); (ii) utilized a retrospective design where outcome awareness could influence predictor assessment, preventing blinding. Other studies (20–26) employed standardized questionnaires and validated clinical tools for predictor collection (e.g., age, depression), resulting in low-risk ratings. (3) Outcomes domain: six studies (21–25, 27) were rated high-risk. Among them, Liu et al. (27) retrospectively extracted both predictors and outcomes, preventing verification of appropriate time intervals between predictor measurement and outcome assessment; five other studies (21–25) introduced measurement bias by implicitly incorporating predictor-related information (e.g., nutritional status, memory scores) into outcome determination. The remaining two studies (20, 26) used standardized outcome definitions and assessments independent of predictors, justifying low-risk ratings. (4) Analysis domain: All eight studies (20–27) were rated high-risk due to multiple limitations: inadequate sample size in one study (22) (events-per-variable [EPV] <10); arbitrary categorization of continuous variables (e.g., diabetes duration) in six studies without clinical justification (21–23, 25–27), and two studies (20, 24) did not report variable handling methods; seven studies (20–24, 26, 27) excluded participants with missing data, risking selection bias; univariable prescreening in seven studies (20, 22–27) ignoring predictor interactions, potentially excluding important multivariable predictors or retaining collinear variables (28, 29); Liu et al. (27) omitted consideration of censoring or competing risks (critical for survival outcomes), while cross-sectional designs (20–26) inherently avoided this limitation; one study (26) reported only AUC without calibration metrics; two studies (26, 27) failed to address overfitting.

#### 3.5.2 Applicability

Regarding the study population, one study (27) exclusively included individuals aged ≥ 80 years, a cohort exhibiting marked differences in physiological functions and comorbidity profiles (e.g., polypharmacy, accelerated cognitive decline rates) compared to those aged 60–79 years (30, 31). Such disparities may reduce the predictive weight of factors (e.g., lifestyle variables) in younger elderly subgroups, thereby limiting the generalizability of findings to the 60–79 age stratum. In contrast, predictors incorporated in the eight studies (20–27) comprehensively covered essential clinical indicators, demonstrating good applicability. Furthermore, all eight studies (20–27) employed internationally consensus-based standards for outcome definitions, which also enhanced their applicability.

### 3.6 Meta-analysis results

The study by Liu et al. (27) was excluded from the pooled analysis due to a logical inconsistency in the validation cohort AUC confidence interval (point estimate < lower bound), confirmed upon verification of the original data. Consequently, only seven studies (20–26) met the eligibility criteria and were included in the meta-analysis.

**Figure 3:**
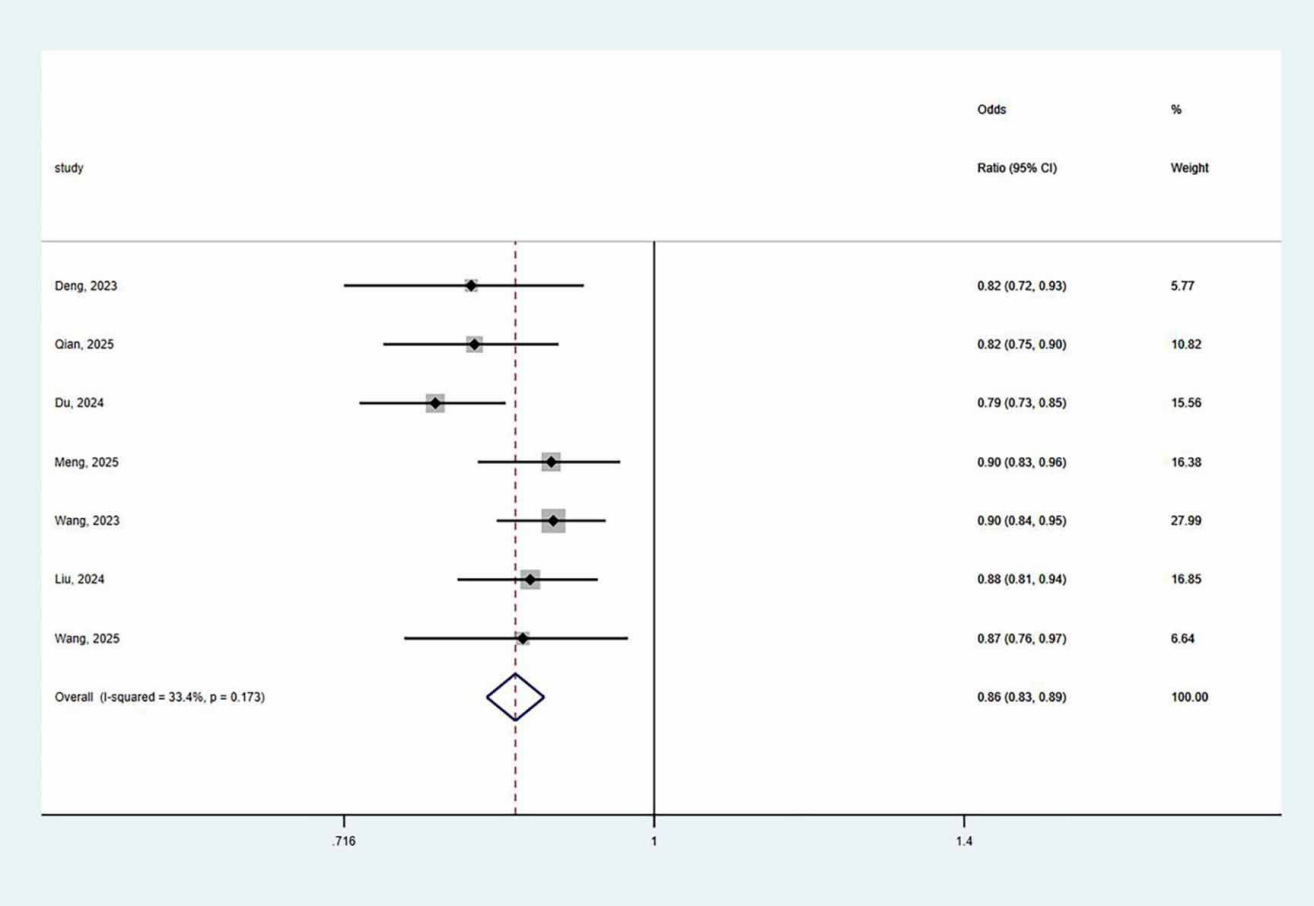
Forest plots of 7 models included.

**Figure 4:**
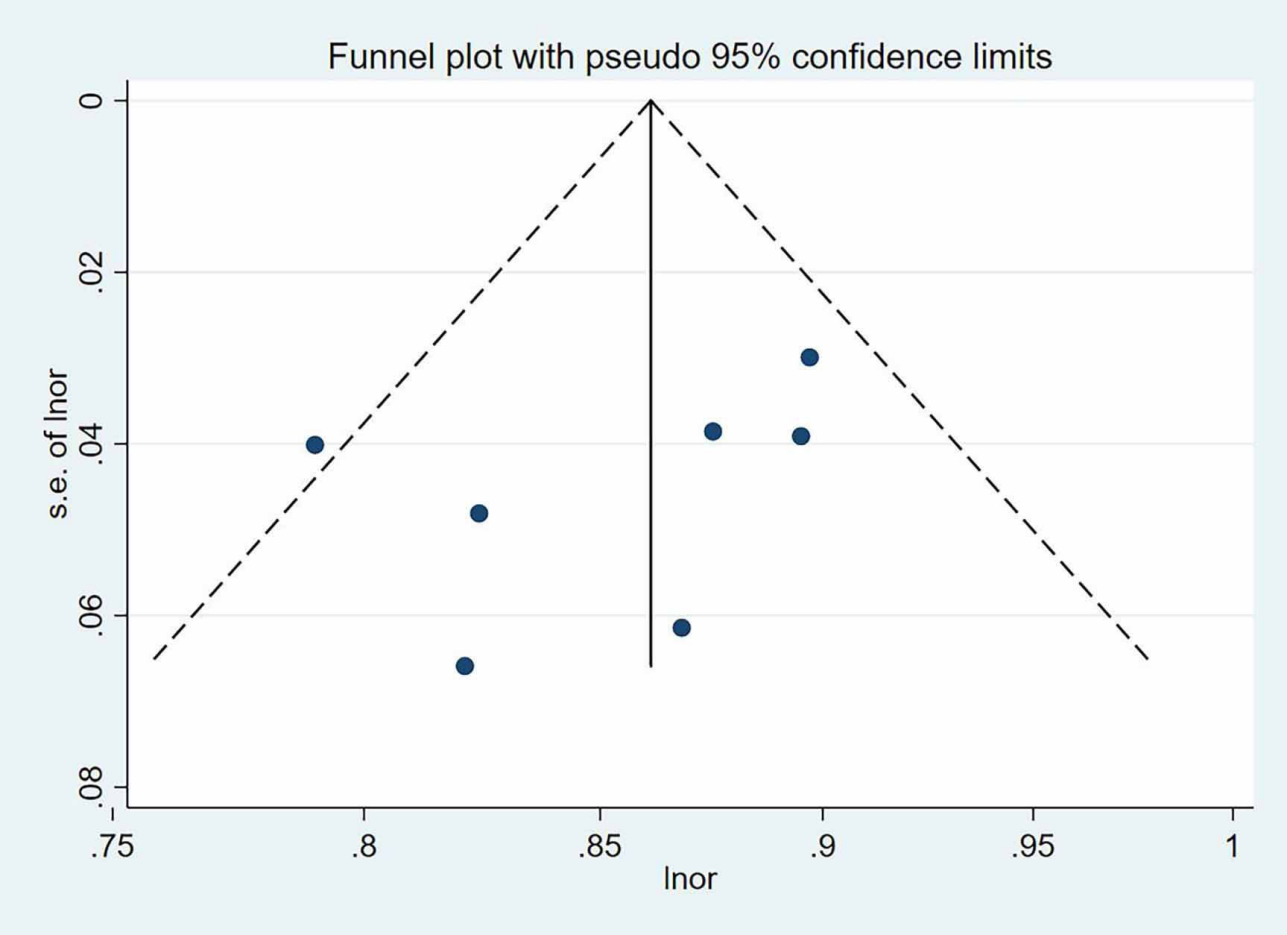
Funnel plot for 7 models included.

Given acceptable heterogeneity (I² = 33.4%, P > 0.1), a fixed-effects model was employed, yielding a pooled AUC of 0.860 (95% CI: 0.830–0.890). This indicates robust overall predictive performance (**Figure 3**). Sensitivity analysis involving sequential exclusion of individual studies, demonstrated stability in the pooled estimate. Furthermore, the funnel plot of the AUC values revealed no significant asymmetry (**Figure 4**), and Egger’s test yielded a non-significant P-value of 0.296 (P > 0.05), suggesting no substantial publication bias.

## 4 Discussion

### 4.1 Models demonstrated generally good predictive performance but with substantial bias risk

This systematic review evaluated eight CF prediction models, which demonstrated moderate-to-excellent predictive performance upon internal or external validation, with AUC values ranging from 0.790 to 0.975. Furthermore, a meta-analysis of seven eligible models yielded a pooled AUC of 0.860 (95% CI: 0.830-0.890), indicating that existing models collectively show good discriminatory ability for assessing CF risk in older adults with diabetes. However, the PROBAST assessment identified a high risk of bias across all included studies, primarily attributable to methodological limitations including: inappropriate study design; outcome definitions failing to exclude the potential influence of predictors; inadequate sample size; arbitrary transformation of continuous variables to categorical formats; suboptimal handling of missing data; reliance on univariable analysis for predictor selection; insufficient model evaluation; and inadequate consideration of model overfitting.

Future research priorities conducting multicenter, large-sample prospective studies that adhere to the PROBAST framework. Key methodological enhancements should focus on: standardizing definitions of predictors and outcomes, assessed by independently trained personnel; employing multiple imputation to reduce the adverse impacts of missing data on statistical analyses and model stability (32); integrating domain knowledge with advanced techniques such as LASSO regression for predictor selection and collinearity handling (33); and ensuring comprehensive reporting of calibration data (a critical measure of agreement between predicted probabilities and actual risk (34)), since its omission increases the risk of bias. Furthermore, while internal validation reduces overfitting and external validation assesses generalizability (35), only two existing studies (21, 23) implemented both approaches. Future work must therefore strengthen external validation across diverse populations (considering geographic, ethnic, cultural, and lifestyle factors) to enhance model applicability.

### 4.2 Consensus on key predictors: age, depression, diabetes duration, nutritional status, and regular exercise

The eight included studies reported 4–8 independent predictors each, broadly categorized into four groups: sociodemographic factors (e.g., age, sex, education level), disease-related factors (e.g., diabetes duration, history of hypoglycemia, chronic kidney disease), psychological factors (e.g., depression), and lifestyle factors (e.g., nutritional status, regular exercise, sleep quality). Despite variations in study design and predictor inclusion, several predictors consistently emerged as most frequent, including age, depression, diabetes duration, nutritional status, and regular exercise.

Research consistently demonstrates that CF risk increases with age (20). Individuals aged ≥ 75 years face a substantially elevated risk, estimated at 5.8-fold higher than those under 65 years (25). This association likely arises from age-related physiological decline, encompassing diminished muscle strength and mass, and atrophy of the hippocampus and cerebral cortex, which directly impairing cognitive reserve and neuroplasticity (5, 36). In diabetic populations, metabolic dysregulation such as hyperglycemia may accelerate these age-associated neurodegenerative processes, thereby promoting CF (27). Furthermore, disease duration and aging represent intertwined risk factors in older diabetic patients. Extended exposure to diabetes heightens cumulative damage to the central nervous system stemming from chronic hyperglycemia, insulin resistance, and microvascular complications. These pathological processes impair vascular endothelial function, provoke chronic cerebrovascular injury, and exacerbate oxidative stress and neuroinflammation (26, 37). Ultimately, sustained metabolic disturbances induce structural brain damage (e.g., hippocampal atrophy, cortical thinning) and functional connectivity impairments, directly driving cognitive decline and the emergence of frailty phenotypes (38).

Concurrently, psychological status, nutrition status, and exercise habits warrant particular attention in diabetic patients (1). Depression and CF exhibit a bidirectional association: Pathologically, depression correlates with dysregulation of the hypothalamic-pituitary-adrenal (HPA) axis, elevated neuroinflammatory cytokines (e.g., IL-6, TNF-α), reduced brain-derived neurotrophic factor (BDNF), and impaired hippocampal neurogenesis—all of these changes directly compromise cognitive function (particularly executive function and memory) and may accelerate cerebral atrophy (39–41). Additionally, depression often manifests with diminished motivation and social withdrawal, which can restrict participation in cognitively beneficial physical activities and social engagement, thereby increasing CF risk (42, 43). (2) Adequate nutrition is critical for sustaining cerebral energy metabolism, synaptic plasticity, neurotransmitter synthesis, and antioxidant defenses. However, diabetic patients face a 21.5% prevalence of malnutrition due to dietary imbalances, glucose utilization impairments, and micronutrient deficiencies (25). Furthermore, malnutrition constitutes a core frailty feature (e.g., weight loss, sarcopenia) and exacerbates cognitive impairment through mechanisms like insufficient energy supply, chronic inflammation, and oxidative stress (44, 45). (3) Regular exercise serves as a protective factor against CF (22). Fink et al. (46) demonstrated a 1.43-fold higher risk of cognitive decline in patients lacking regular exercise habits compared to those engaging in either moderate-intensity activity (> 30 minutes, ≥ 5 days/week) or high-intensity activity (> 20 minutes, ≥ 3 days/week). Mechanistically, exercise combats CF by enhancing insulin sensitivity and glucose metabolism, promoting muscle protein synthesis and delaying bone loss, and improving cerebral plasticity and cerebral blood flow (47).

Therefore, it is recommended to prioritize older adults with diabetes (particularly those aged ≥ 75 years or with longer disease duration) as high-risk populations for CF requiring intensive monitoring. Key predictor assessment and intervention should be systematically integrated into routine diabetes management: implement regular depression screening using validated tools like the PHQ-946 (48), initiating timely psychological interventions when indicated; assess nutritional risk with instruments such as the MNA-SF47 (49), dynamically monitoring weight, appetite, eating capacity, and dietary patterns, while providing tailored nutritional guidance; systematically record physical activity frequency, intensity, and duration to identify sedentary individuals or those with functional impairments, developing personalized exercise prescriptions based on baseline fitness, comorbidities (e.g., arthritis, cardiovascular disease), patient preferences, and environmental factors.

The consensus on key predictors provides an essential foundation for model development. For predictive models to effectively inform clinical decision-making, however, clinicians must be able to rapidly assess their applicability to specific practice contexts. The PICOTS framework offers a systematic approach to defining a model’s target population, predictors, outcome definition, time horizon, and setting (18). For example, clear reporting of outcome definitions (e.g., diagnostic criteria for CF) and prediction timeframes (e.g., 1−year vs. 5−year risk) allows clinicians to evaluate alignment with management goals. Likewise, detailed descriptions of population characteristics (e.g., age range, diabetes type) and clinical settings (e.g., primary or specialty care) facilitate evaluation of generalizability to target populations. Thus, in addition to methodological improvements, future studies should emphasize comprehensive, PICOTS−guided reporting. This will help clinicians efficiently identify the most applicable prediction tools, thereby advancing evidence−based, individualized risk assessment and management.

### 4.3 Limitations of this study

This study has several inherent limitations. First, while the analysis focused on evaluating the predictive performance and bias risk of included models, it did not directly verify their applicability in real-world clinical settings. Second, although the review was guided by the PICOTS framework, certain elements such as the timing of outcome assessment and prediction horizons were often inadequately reported, limiting our capacity for detailed subgroup analyses and comparative synthesis. Third, as the findings primarily derived from Chinese patient data and 75% of the included studies lacked external validation, the generalizability of the results may be limited. Finally, restricting the literature search to Chinese and English publications could have introduced selection bias.

## 5 Conclusion

This study synthesized and critically evaluated existing prediction models for CF risk in older adults with diabetes. The results indicate that while current models demonstrate satisfactory discriminatory performance, their overall methodological quality requires improvement. Key limitations include insufficient external validation and clinical application studies, as well as limited evidence regarding their real-world clinical utility. Consequently, future research should rigorously adhere to established methodological and reporting guidelines to enhance the development of robust risk prediction models. Furthermore, successful translation of these models into practical digital risk alert systems is essential to deliver evidence-based tools for CF prevention in this vulnerable population.

## Data Availability

The original contributions presented in the study are included in the article/**Supplementary Material**. Further inquiries can be directed to the corresponding author.
