# Supplementary material for: Risk prediction model for cognitive frailty in older adults with diabetes: a systematic review and meta-analysis
Source: Front Endocrinol (Lausanne). 2026 Apr 10;17:1794278. doi: 10.3389/fendo.2026.1794278 (PMC13105987; doi:10.3389/fendo.2026.1794278)
Supplement: Supplementary file 1 [file Table1.docx]

| **Table S1.** Search Strategy for Each Database. | |
| --- | --- |
| **1.PubMed** | |
| #1 | "Diabetes Mellitus"[MeSH Terms] OR "Diabetes"[Title/Abstract] OR "Diabetic"[Title/Abstract] |
| #2 | "cognitive frailty"[Title/Abstract] OR "cognitive dysfunction*"[Title/Abstract] OR "cognitive decline*"[Title/Abstract] OR "cognitive impairment*"[Title/Abstract] OR "cognitive disorder*"[Title/Abstract] OR "Frailty"[Title/Abstract] OR "Frailties"[Title/Abstract] OR "frailty syndrome"[Title/Abstract] OR "Debility"[Title/Abstract] OR "Debilities"[Title/Abstract] OR "Asthenia"[Title/Abstract] OR "Frail"[Title/Abstract] |
| #3 | "predict*"[Title/Abstract] OR "predict* model"[Title/Abstract] OR "risk assessment"[Title/Abstract] OR "risk score"[Title/Abstract] OR "risk prediction"[Title/Abstract] OR "risk factor"[Title/Abstract] OR "Forecast"[Title/Abstract] OR "Model"[Title/Abstract] OR "Nomogram"[Title/Abstract] OR "area under curve"[Title/Abstract] |
| #4 | #1 AND #2 AND #3 |
|  | |
| **2.Embase** | |
| #1 | diabetes mellitus'/exp OR 'diabetes':ab,ti OR 'diabetic':ab,ti |
| #2 | cognitive frailty':ab,ti OR 'cognitive dysfunction*':ab,ti OR 'cognitive decline*':ab,ti OR 'cognitive impairment*':ab,ti OR 'cognitive disorder*':ab,ti OR 'frailty':ab,ti OR 'frailties':ab,ti OR 'frailty syndrome':ab,ti OR 'debility':ab,ti OR 'debilities':ab,ti OR 'asthenia':ab,ti OR 'frail':ab,ti |
| #3 | predict*':ab,ti OR 'predict* model':ab,ti OR 'risk assessment':ab,ti OR 'risk score':ab,ti OR 'risk prediction':ab,ti OR 'risk factor':ab,ti OR 'forecast':ab,ti OR 'model':ab,ti OR 'nomogram':ab,ti OR 'area under curve':ab,ti |
| #4 | #1 AND #2 AND #3 |
|  | |
| **3.Web of SScience** | |
| #1 | TS=(Diabetes Mellitus OR Diabetes OR Diabetic) |
| #2 | TS=(Cognitive frailty OR Cognitive dysfunction* OR Cognitive decline* OR Cognitive impairment* OR Cognitive disorder* OR Frailty OR Frailties OR Frailty Syndrome OR Debility OR Debilities OR Asthenia OR Frail) |
| #3 | TS=(Predict* OR Predict* model OR Risk assessment OR Risk score OR Risk prediction OR Risk factor OR Forecast OR Model OR Nomogram OR Area under curve) |
| #4 | #1 AND #2 AND #3 |
|  | |
| **4.The Cochrane Library** | |
| #1 | MeSH descriptor: [Diabetes Mellitus] explode all trees |
| #2 | (Diabetes):ti,ab,kw OR (Diabetes):ti,ab,kw |
| #3 | #1 OR #2 |
| #4 | (Cognitive frailty):ti,ab,kw OR (Cognitive dysfunction*):ti,ab,kw OR (Cognitive decline*):ti,ab,kw OR (Cognitive impairment*):ti,ab,kw OR (Cognitive disorder*):ti,ab,kw |
| #5 | (Frailty):ti,ab,kw OR (Frailties):ti,ab,kw OR (Frailty Syndrome):ti,ab,kw OR (Debility):ti,ab,kw OR (Debilities):ti,ab,kw |
| #6 | (Asthenia):ti,ab,kw OR (Frail):ti,ab,kw |
| #7 | #4 OR #5 OR #6 |
| #8 | (Predict*):ti,ab,kw OR (Predict* model):ti,ab,kw OR (Risk assessment):ti,ab,kw OR (Risk score):ti,ab,kw OR (Risk prediction):ti,ab,kw |
| #9 | (Risk factor):ti,ab,kw OR (Forecast):ti,ab,kw OR (Model):ti,ab,kw OR (Nomogram):ti,ab,kw OR (Area under curve):ti,ab,kw |
| #10 | #8 OR #9 |
| #11 | #3 AND #7 AND #10 |
|  | |
| **5.CINAHL** | |
| #1 | XB (Diabetes Mellitus OR Diabetes OR Diabetic) |
| #2 | XB (Cognitive frailty OR Cognitive dysfunction* OR Cognitive decline* OR Cognitive impairment* OR Cognitive disorder* OR Frailty OR Frailties OR Frailty Syndrome OR Debility OR Debilities OR Asthenia OR Frail) |
| #3 | XB (Predict* OR Predict* model OR Risk assessment OR Risk score OR Risk prediction OR Risk factor OR Forecast OR Model OR Nomogram OR Area under curve) |
| #4 | #1 AND #2 AND #3 |
|  | |
| **6.Sinomed** | |
| #1 | "糖尿病"[主题词] OR "糖尿病"[常用字段] |
| #2 | "认知衰弱"[常用字段] OR "认知障碍"[常用字段] OR "认知下降"[常用字段] OR "认知损害"[常用字段] OR "认知减退"[常用字段] OR "衰弱"[常用字段] OR "衰弱综合征"[常用字段] OR "虚弱"[常用字段] |
| #3 | "预测"[常用字段] OR "预测因子"[常用字段] OR "预测模型"[常用字段] OR "风险评估"[常用字段] OR "风险评分"[常用字段] OR "风险预测"[常用字段] OR "风险因素"[常用字段] OR "模型"[常用字段] OR "列线图"[常用字段] OR "曲线下面积"[常用字段] |
| #4 | #1 AND #2 AND #3 |
|  | |
| **7.CNKI** | |
| (主题:糖尿病)AND(主题:认知衰弱 OR 认知障碍 OR 认知下降 OR 认知损害 OR 认知减退 OR 衰弱 OR 衰弱综合征 OR 虚弱)AND(主题:预测 OR 预测因子 OR 预测模型 OR 风险评估 OR 风险评分 OR 风险预测 OR 风险因素 OR 模型 OR 列线图 OR 曲线下面积) | |
|  | |
| **8.Wanfang** | |
| 主题:(糖尿病) and 主题:(认知衰弱 OR 认知障碍 OR 认知下降 OR 认知损害 OR 认知减退 OR 衰弱 OR 衰弱综合征 OR 虚弱) and 主题:(预测 OR 预测因子 OR 预测模型 OR 风险评估 OR 风险评分 OR 风险预测 OR 风险因素 OR 模型 OR 列线图 OR 曲线下面积) | |
